# Concerted evolution and unorthodox recombination of human subtelomeres

**DOI:** 10.64898/2026.07.10.737660

**Published:** 2026-07-10

**Authors:** Andrea Guarracino, Angela Gyamfi, Human Pangenome Reference Consortium, Erik Garrison

## Abstract

Human subtelomeres contain duplicated sequence that is shared among the ends of non-homologous chromosomes and provides a substrate for ectopic exchange [1–6]. However, incomplete reference assemblies and chromosome-by-chromosome analyses have prevented a population-scale view of the extent and organization of subtelomeric exchange [7–9]. Here we apply a reference-free pangenome approach to 465 near-complete human assemblies, comparing every chromosome end against every other, and find that high-identity pseudo-homolog regions occur on 41 of 48 chromosome arms. These regions form structured sequence communities in which previously described exchange systems appear as local peaks within a broader continuum. Human and mouse chromosome-contact maps show preferential proximity between subtelomeres with similar sequences. In mouse meiosis this proximity is strongest at the zygotene bouquet, when telomeres cluster at the nuclear envelope; in human data it persists even in adjacent flanks that lack the shared sequence used to define each pair. In a three-generation telomere-to-telomere pedigree, whole-genome comparison identifies putative recombination between subtelomeric regions on non-homologous chromosomes that matches this community organization, while recovering the obligate Xp/Yp PAR1 recombination in the male germline. These results generalize known subtelomeric exchange systems into a near-ubiquitous architecture and support recurrent ectopic exchange as a genome-wide force in the concerted evolution of human chromosome ends.

## Full Text Availability

The license terms selected by the author(s) for this preprint version do not permit archiving in PMC. The full text is available from the preprint server.

